# Dataset of audio signals from brushless DC motors for predictive maintenance

**DOI:** 10.1016/j.dib.2023.109569

**Published:** 2023-09-13

**Authors:** Rommel Stiward Prieto Estacio, Diego Alberto Bravo Montenegro, Carlos Felipe Rengifo Rodas

**Affiliations:** Universidad del Cauca, Calle 5 Nro. 4-70, Popayán (Cauca), Colombia

**Keywords:** Machine learning, Audio analysis, BLDC motors, Predictive maintenance

## Abstract

Predictive Maintenance (**PdM**) has a main role in the Fourth Industrial Revolution; its goal is to design models that can safely detect failure in systems before they fail, aiming to reduce financial, environmental, and operational costs. A brushless DC (BLDC) electric motors have increasingly become more popular and been gaining popularity in industrial applications, so their analysis for **PdM** applications is only a natural progression; audio analysis proves to be a useful method to achieve this and rises as a very pragmatic case of study of the characteristics of the motors. The main goal of this paper is to showcase sound-based behavior of BLDC motors in different failure modes as result of an experiment led by researchers at Universidad del Cauca in Colombia. This dataset may provide researchers with useful information regarding signal processing and the development of Machine Learning applications that would achieve an improvement within Predictive Maintenance and I4.0.Predictive Maintenance (**PdM**) has a main role in the Fourth Industrial Revolution; its goal is to design models that can safely detect failure in systems before they fail, aiming to reduce financial, environmental, and operational costs. A brushless DC (BLDC) electric motors have increasingly become more popular and been gaining popularity in industrial applications, so their analysis for **PdM** applications is only a natural progression; audio analysis proves to be a useful method to achieve this and rises as a very pragmatic case of study of the characteristics of the motors. The main goal of this paper is to showcase sound-based behavior of BLDC motors in different failure modes as result of an experiment led by researchers at Universidad del Cauca in Colombia. This dataset may provide researchers with useful information regarding signal processing and the development of Machine Learning applications that would achieve an improvement within Predictive Maintenance and I4.0.

Specifications Table

**Table:** 

Subject	Electrical and Electronic Engineering.
Specific subject area	Audio-based maintenance of Brushless DC Motors
Data format	Raw (.wav format)
Type of data	Raw data in .wav format separated by fault categories and motors.
Data collection	This dataset contains 43 .wav files of approximately 10 s each, with a 16 kHz sampling frequency containing the sound of four A2212 BLDC motors submitted to different categories: healthy motors, propeller failure and bearing failure. These audio files may be useful for signal processing and Machine Learning applications in Predictive Maintenance [1]. Data was obtained by connecting BLDC motors to a 11.1 LiPo battery, a 30 A ESC, and an ESP32 to control the motor's speed via Blynk to obtain the motors sound, which were recorded at 16 kHz using a MCJR-005 Capacitor Microphone.
Data source location	Institution: Universidad del Cauca City/Region: Popayán, Cauca Country: Colombia
Data accessibility	Repository name: Mendeley Data Data identification number: 10.17632/j4yr5fmhv4.1 Direct URL to data: https://data.mendeley.com/datasets/j4yr5fmhv4/1

## 1 Value of the Data


 

• This data provides a collection of audio signals emitted by A2212 Brushless DC motors submitted to different status: healthy, propeller fault, and bearing fault, [1].
• The dataset might be useful for researchers and Machine Learning developers as part of feature extraction, signal processing and design of machine learning models for predictive maintenance applications, [[2], [3]].
• Researchers are expected to find this dataset suitable for classification and regression models after performing satisfactory feature extraction.

## 2 Data Description

The dataset consists of .wav files ordered as follows:

- BLDC_sound_data
  ○ Bearing
    ■ M4
      • 1700.wav
      • 1750.wav
      • 1800.wav
      • 1850.wav
      • 1900.wav
      • 1950.wav
      • 2000.wav
  ○ Healthy
    ■ M1
      • 1400.wav
      • 1450.wav
      • 1550.wav
      • 1650.wav
      • 1750.wav
      • 1850.wav
      • 1950.wav
      • 2000.wav
    ■ M2
      • 1400.wav
      • 1450.wav
      • 1550.wav
      • 1650.wav
      • 1750.wav
      • 1850.wav
      • 1950.wav
      • 2000.wav
    ■ M3
      • 1450.wav
      • 1550.wav
      • 1650.wav
      • 1750.wav
      • 1850.wav
      • 1950.wav
  ○ Propeller
    ■ M1
      • 1400.wav
      • 1450.wav
      • 1550.wav
      • 1650.wav
      • 1750.wav
      • 1850.wav
    ■ M2
      • 1400.wav
      • 1450.wav
      • 1550.wav
      • 1650.wav
      • 1750.wav
      • 1850.wav
      • 1950.wav
      • 2000.wav

Each .wav file is approximately 10 s long, so during pre-processing files must be trimmed to 10 s to properly analyze them. Files were recorded at 16 kHz frequency, so once they have been loaded and trimmed each file should be stored in a 160,000 × 1 array.

## 3 Experimental Design, Materials and Methods

**Fig. 1:**
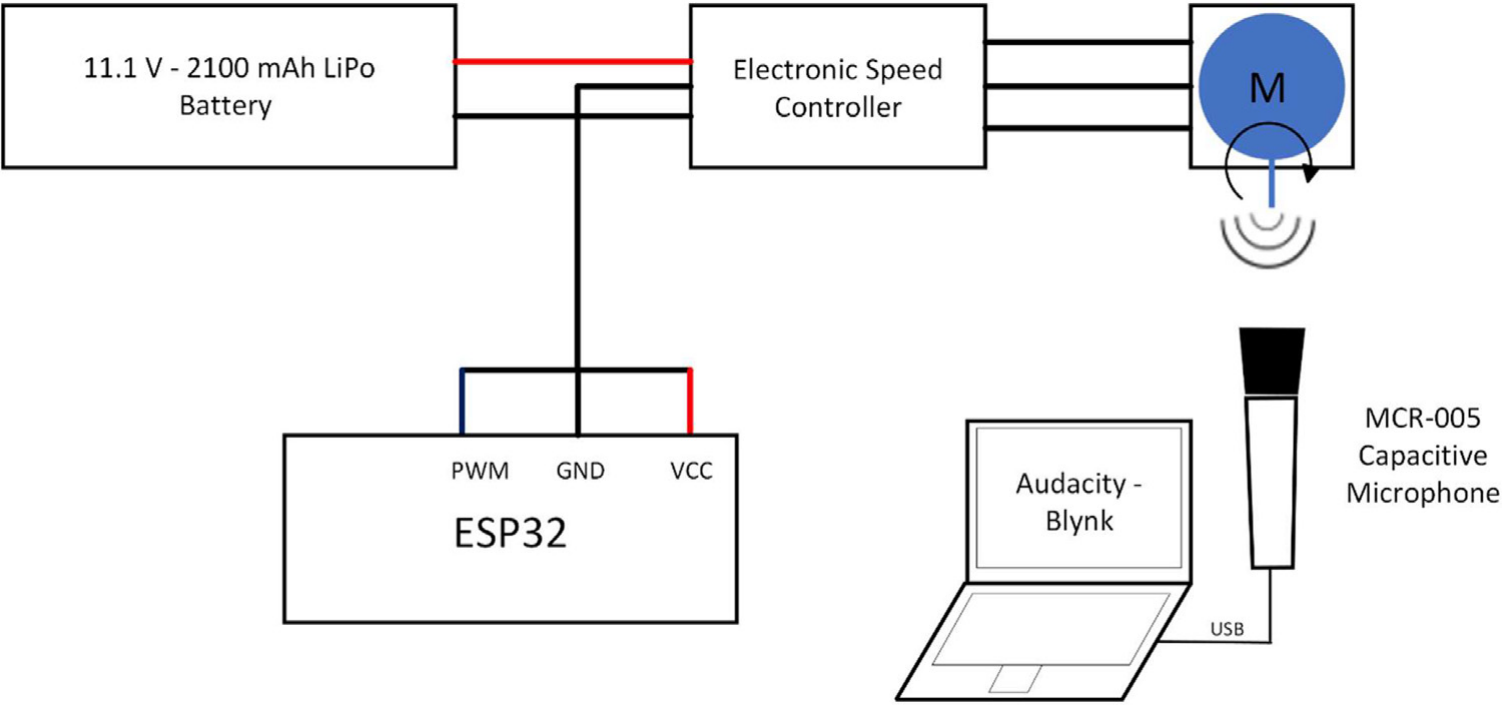
Experimental setup for data acquisition.

The dataset contains 43 recordings of four different A2212 Brushless DC motors under different operation modes. Motors 1 and 2 were submitted to both normal circumstances and broken propeller fault, while motor 3 and 4 provide information only for the healthy and bearing fault categories respectively. Each motor was connected to a 30 A Electronic Speed Controller, supplied by a 11.1 V-2100 mAh LiPo battery; the speed of the motor was controlled using an ESP32, and this procedure can be performed in two manners: the first, is by reading a potentiometer and writing its value to the ESC, and the second, more IoT oriented is by connecting the ESP32 to a Blynk server, using a slider to write a value to the ESC. This second method offers more stability and control over the intervals but also is very dependent on the internet latency. Ten seconds of audio at 16 kHz are recorded using Audacity as an open-source software and the speed of the motors is incremented a fixed amount according to the category. Fig. 1 provides a scheme of the experimental setup used to obtain the audio recordings.

**Fig. 2:**
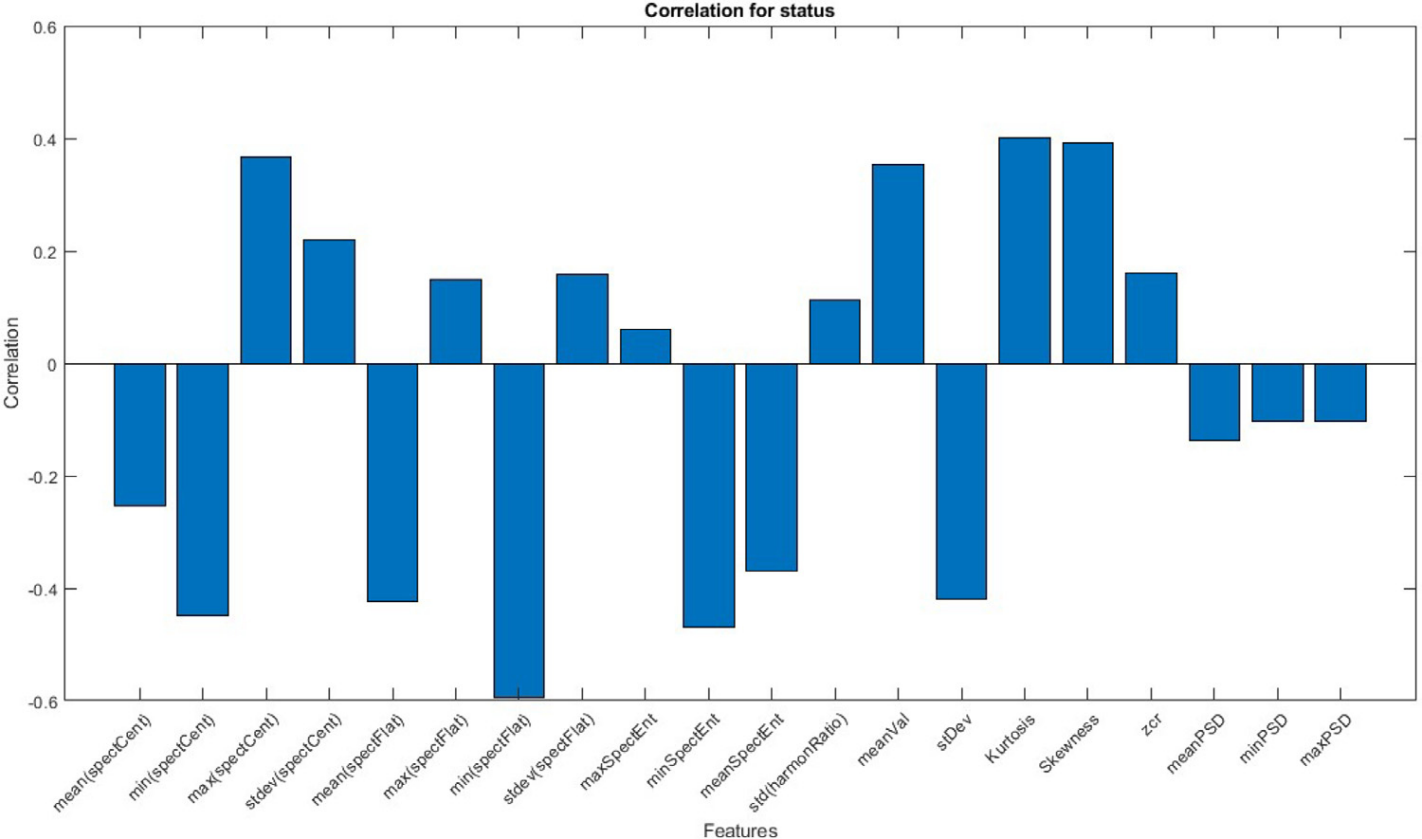
Pearson correlation graph between 20 features and 3 classes.

The data is then properly classified, and a Pearson correlation analysis is performed to determine which combination of statistical and spectral features could be useful to train a Machine Learning model that can predict the current operational mode of a BLDC motor. Fig. 2 shows the result of said correlation analysis. Readers of this work are expected to download the audio files (*.wav) [3] and perform digital audio processing to extract the features presented in Fig. 2.

## Limitations

The data was collected in a controlled environment minimizing outside noise.

## Ethics Statement

The production of the data collected did not involve any human subjects, animal experimentation, nor any data from social media platforms. The authors have read and follow the ethical requirements for publication in Data in Brief.

## CRediT authorship contribution statement

**Rommel Stiward Prieto Estacio:** Conceptualization, Methodology, Software. **Diego Alberto Bravo Montenegro:** Visualization, Investigation. **Carlos Felipe Rengifo Rodas:** Writing – review & editing.

## Data Availability

Brushless DC Motor sound dataset for PdM (Original data) (Mendeley Data).
